# Cost–benefit analysis of beach-cast harvest: Closing land-marine nutrient loops in the Baltic Sea region

**DOI:** 10.1007/s13280-021-01641-8

**Published:** 2021-11-17

**Authors:** Tore Söderqvist, Hanna Nathaniel, Daniel Franzén, Frida Franzén, Linus Hasselström, Fredrik Gröndahl, Rajib Sinha, Johanna Stadmark, Åsa Strand, Ida Ingmansson, Sofia Lingegård, Jean-Baptiste Thomas

**Affiliations:** 1 Holmboe & Skarp AB, Norr Källstavägen 9, 148 96 Sorunda, Sweden; 2 Department of Sustainable Development, Environmental Science and Engineering, KTH Royal Institute of Technology, Teknikringen 10B, 100 44 Stockholm, Sweden; 3 Tyréns AB, Peter Myndes Backe 16, 118 46 Stockholm, Sweden; 4 IVL Swedish Environmental Research Institute, Box 53021, 400 14 Göteborg, Sweden; 5 IVL Swedish Environmental Research Institute, Kristineberg 566, 451 78 Fiskebäckskil, Sweden

**Keywords:** Beach recreation, Beach wrack, Bioeconomy, Circular economy, Eutrophication, Nutrient loops

## Abstract

Harvesting beach-cast can help mitigate marine eutrophication by closing land-marine nutrient loops and provide a blue biomass raw material for the bioeconomy. Cost–benefit analysis was applied to harvest activities during 2009–2018 on the island of Gotland in the Baltic Sea, highlighting benefits such as nutrient removal from the marine system and improved recreational opportunities as well as costs of using inputs necessary for harvest. The results indicate that the activities entailed a net gain to society, lending substance to continued funding for harvests on Gotland and assessments of upscaling of harvest activities to other areas in Sweden and elsewhere. The lessons learnt from the considerable harvest experience on Gotland should be utilized for developing concrete guidelines for carrying out sustainable harvest practice, paying due attention to local conditions but also to what can be generalized to a wider national and international context.

**Supplementary Information:**

The online version contains supplementary material available at 10.1007/s13280-021-01641-8.

## Introduction

The biogeochemical flows of nitrogen (N) and phosphorus (P) are judged to exceed planetary boundaries (Folke et al. 2021). Excessive anthropogenic nutrient-loading causes effects such as severe eutrophication and deoxygenation in coastal oceans (Diaz and Rosenberg 2008), and one of the world’s largest dead zones is found in the Baltic Sea (Carstensen et al. 2014), which also suffer from other eutrophication mediated effects (e.g., Rönnbäck et al. 2007; Snickars et al. 2015). This also affects people’s wellbeing; the public has repeatedly communicated a willingness to pay for improved environmental conditions such as clearer waters, cleaner beaches and reduced risks for algal blooms (see “Nutrient removal from the marine system” section).

While land-based measures have decreased N and P emissions (Andersen et al. 2017), N and P loads to the sea are still substantial (HELCOM 2018), exacerbating N and P stock accumulation. Suggested further action such as cleaner production and end-of-pipe approaches (Barquet et al. 2020) does not, however, address one of the main reasons for why Baltic Sea conditions remain poor, i.e., the legacy of earlier emissions, notably of legacy P accumulated in sediments (McCrackin et al. 2018; Limburg et al. 2020). This motivates complementary measures that assimilate and recycle nutrients that have already entered marine environments, e.g., harvest of beach-cast (Gröndahl et al. 2009; Quilliam et al. 2015; Chubarenko et al. 2021). As indicated by a life-cycle assessment by Thomas et al. (2021), harvesting beach-cast could contribute to closing land-marine nutrient loops, thus mitigating further nutrient accumulation and reducing legacy nutrients stocks. Harvests could also reduce the detrimental effects of excessive beach-cast, a phenomenon present in the Baltic Sea and many other coastal regions worldwide (Smetacek and Zingone 2013; Weinberger et al. 2020).

**Fig. 1:**
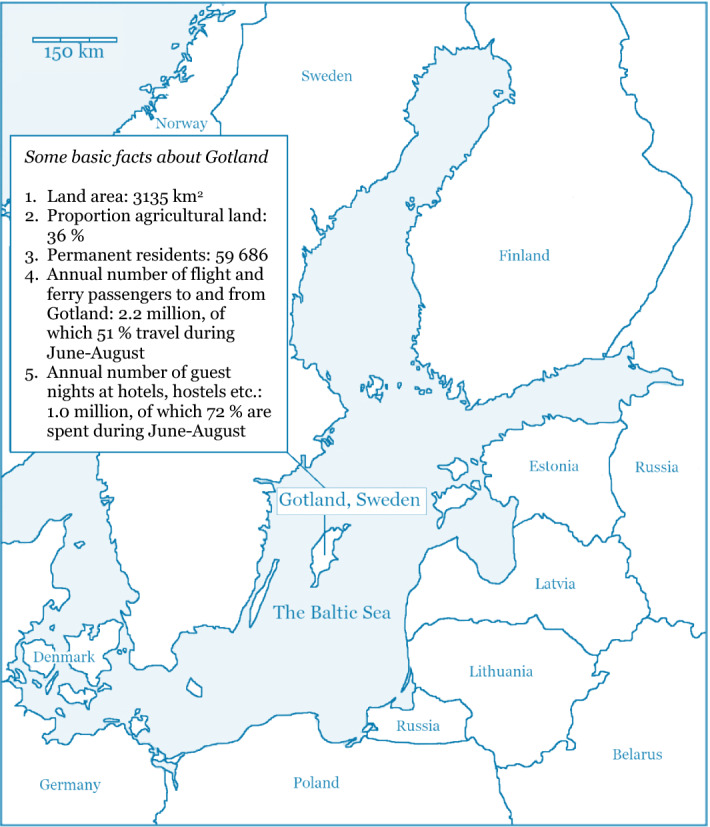
The island of Gotland in the Baltic Sea, and some facts highlighting the island’s large proportion of agricultural land and its attractiveness for tourists during summers. Fact items 1–2 are for 2015, 3 is for 31 December 2019, and 4–5 are for 2018. Sources: Statistics Sweden (2021a) for 1–3, Region Gotland (2019) for 4, and Statistics Sweden (2021b) for 5

Beach-cast harvest has traditionally been carried out for fertilization and soil improvement in agriculture in coastal Baltic Sea provinces such as the island of Gotland (Franzén et al. 2019). Although current mainstream agricultural practices do not include the use of beach-cast, harvesting has now re-emerged as a measure for curbing eutrophication (SwAM 2019) and in line with the ambitions of circular economy (Geissdoerfer et al. 2017) and blue bioeconomy (Mulazzani and Malorgio 2017; COM 2018). However, beach-cast harvesting also involves costs due to inputs such as labour and machinery, and it entails negative environmental effects such as drift line habitat loss for some plants and invertebrates (Zielinski et al. 2019). By explicitly addressing trade-offs associated with beach-cast harvesting, the focus of this paper is to explore if such harvesting is economically motivated from a societal point of view. That is, are the social benefits of beach-cast harvesting likely to exceed its social costs? We therefore apply cost–benefit analysis (CBA) on beach-cast harvest initiatives, and the results are used to develop recommendations for future beach-cast management. The focus is the situation on Gotland, the largest island in the Baltic Sea, which has beaches that make it a tourism hotspot during the summer (Fig. 1). To our knowledge and as indicated by the summary of earlier literature in Appendix S1, this is the first CBA application on beach-cast harvesting in a marine eutrophication and bioeconomy context, taking into account the nutrient contents of beach-cast and using primary cost data from several similar harvest initiatives. Marine eutrophication and excessive amounts of beach-cast are globally widespread, making these results relevant not only for local management but also in an international context.

## Theoretical framework

Cost–benefit analysis (CBA) relies on the anthropocentric approach of mainstream welfare economics for assessing positive and negative consequences (benefits and costs) of a project in terms of how human wellbeing is affected relative to a reference alternative (baseline) (Johansson and Kriström 2018). The project is economically profitable for society if its total benefits are greater than its total costs. A CBA can be conducted *ex ante* (before a decision about undertaking a project), *ex post* (after a project is completed) or *in medias res* (for an ongoing project) and is typically described as a stepwise procedure, which will be referred to in the following: (1) Explain the purpose; (2) Specify the project(s) to be assessed including the reference alternative; (3) Specify standing, i.e. whose benefits and costs are to be counted; (4) Identify consequences and select metrics; (5) Quantify consequences; (6) Monetize consequences; (7) Discount benefits and costs to obtain present values; (8) Compute net present values; (9) Perform sensitivity analysis; (10) Make a recommendation (Boardman et al. 2018).

**Fig. 2:**
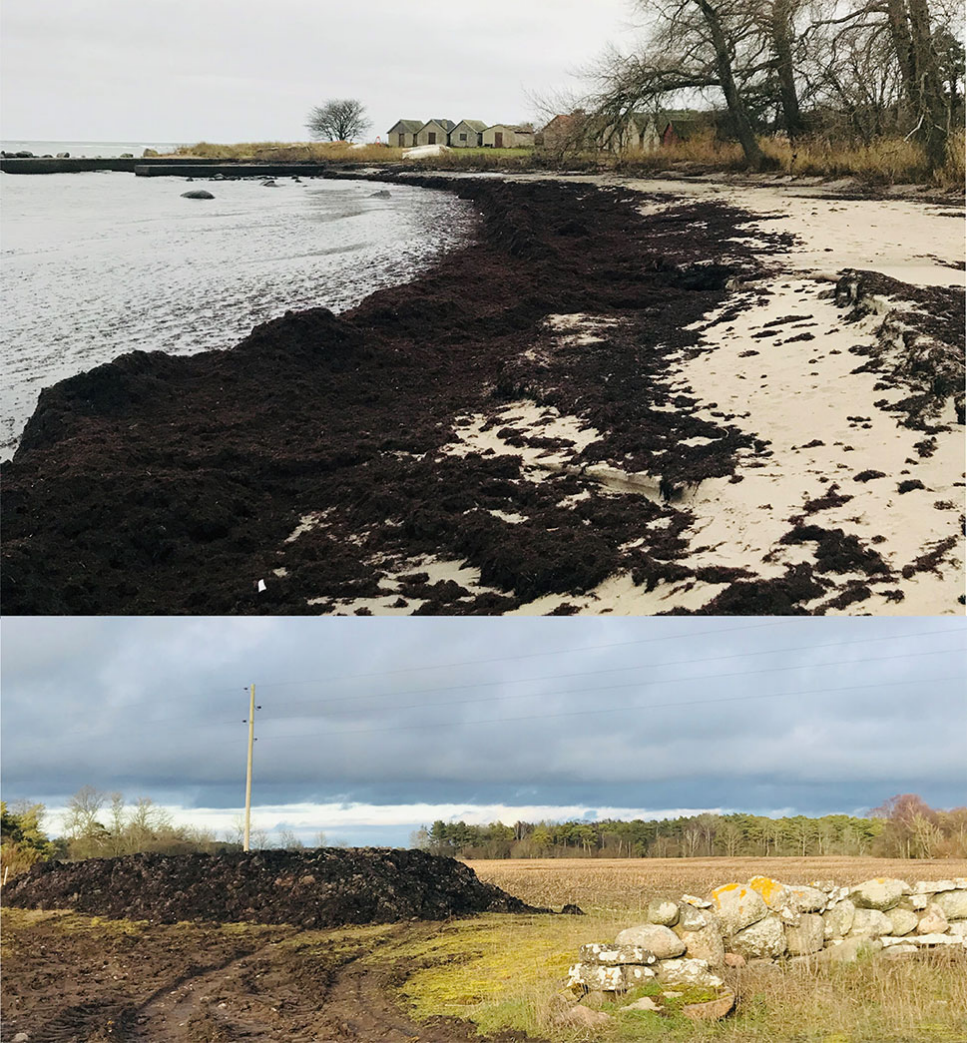
Bands of beach-cast at Norbods fiskeläge, Augstens, southern Gotland (https://goo.gl/maps/26DAaEo4jWHVvU8c9), and a pile of beach-cast placed at adjacent agricultural land. Photos: Hanna Nathaniel, November 2019 and April 2020, respectively

Given the purpose of the CBA as described in “Introduction” section (Step 1), the project to be assessed *ex post* in this paper is defined as recent and completed beach-cast harvest activities carried out on Gotland, and the reference alternative is defined as a situation without those beach-cast harvest activities (Step 2). Harvest refers to moving beach-cast from the shores to piles for decomposition (Fig. 2), see “Method and material” section for details. The potential use of beach-cast is *not* included as a part of the project; see “Discussion” section for a discussion of potential uses. Beach-cast harvest can be expected to affect the wellbeing locally (people living on or visiting Gotland) but also a wider population in the Baltic Sea region because of nutrient removal from the marine system as a whole. A regional approach to standing is therefore applied (Step 3), but a delimitation to the Swedish population is discussed in “Discussion” section given that beach-cast harvests on Gotland are carried out in a Swedish environmental policy context. The results of steps 4–10 are reported in “Results” section. Monetization (Step 6) is applied whenever possible. If data scarcity precludes monetization, consequences are instead assessed at least qualitatively. Given the *ex post* nature of the CBA, monetized benefits and costs occurring at different points in past time are adjusted for inflation through the consumer price index, i.e., expressing them for a common price year.

## Materials and methods

Beach-cast harvesting on Gotland has largely been carried out as local water protection projects (Swedish: *Lokala vattenvårdsprojekt*, LOVA), with project costs partly being covered by national governmental grants administrated by the County Administrative Board of Gotland (CABG). During the period of 2009–2018, 40 LOVA beach-cast harvest projects were carried out on Gotland. These 40 projects constitute together the harvest activities to be assessed *ex post* by the CBA.

Project owners included Gotland Municipality, but most of them were non-governmental organizations (NGOs) such as local community associations focusing on a specific beach. The CBA is based on results reported by these projects to CABG, including harvest costs and nutrient removal estimates based on chemical analysis. To account for uncertainties inferred by the self-reporting of results by project owners, the results are compared to other sources of information about harvest costs and nutrient concentrations in beach-cast. Besides results from earlier literature, complementary data were obtained from semi-structured interviews carried out in another research project in December 2019–May 2020 with 18 stakeholders of Gotland-based beach-cast harvesting activities, representing six NGOs, six actors in the public sector and six actors in the private sector (Appendix S2a). After 2018, additional LOVA projects have been conducted but are not included in this CBA.

In the CBA, benefits and costs are expressed as total monetary amounts for all the 40 LOVA projects during the whole period of 2009–2018, if not otherwise stated. All monetized benefits and costs are expressed in US dollars and 2018 prices (denoted by USD_2018_ in the following), if not otherwise stated. Relevant currency rates and, for benefits and costs that occurred before 2018, consumer price indices were applied, see Appendix S4–S7 for details. The monetization of the benefits of nutrient removal is based on earlier valuation studies of such benefits. Recent and peer-reviewed studies of the benefits of reducing eutrophication effects specifically in the Baltic Sea were selected for maximizing quality and relevance of the results (Appendix S4).

## Results

**Fig. 3:**
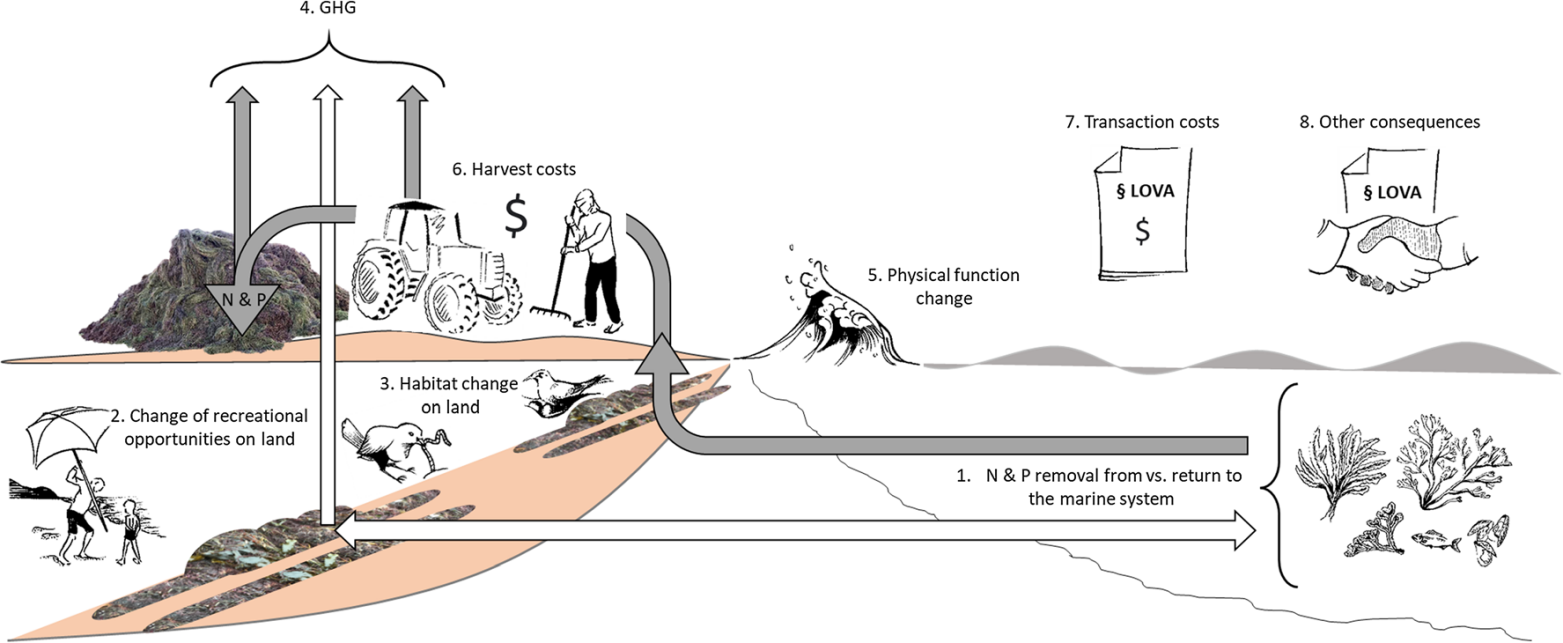
Overview of consequences of relevance for people’s wellbeing due to harvesting and piling beach-cast: Removal of nutrients from the marine system (1, grey arrow), changes in recreational opportunities and habitats on land (2, 3), greenhouse gas (GHG) emissions from piles of beach-cast and harvest machinery (4, grey arrows), change in physical functions such as wave energy reduction (5), harvest costs (6) and transaction costs associated with the LOVA system (7). Other consequences (8) include the local cooperation and knowledge building taking place in the LOVA projects. The white arrows refer to the reference alternative when bands of beach-cast are not harvested; this implies that nutrients are largely returned from beach-cast to the marine system and that GHGs are released from the decomposing beach-cast

Eight main categories of consequences of relevance for people’s wellbeing related to beach-cast harvest on Gotland were identified, see Fig. 3.

### Nutrient removal from the marine system

The total annual harvest of beach-cast on Gotland has been estimated to 12 000–15 000 metric tons (t) fresh weight (FW) (Ulf Smedberg, pers. comm. 3 December 2018; see also Appendix S5), which suggests that LOVA projects during the 10-year period in question (2009–2018) have accounted for 60–75% of the total beach-cast and associated nutrient removal on Gotland (Table 1; see Appendix S3 for similar results based on an alternative approach).

**Table 1:** Total beach-cast harvest and nutrient removal from the Baltic Sea, based on the projects’ chemical analyses of beach-cast samples accomplished by LOVA projects during 2009–2018. Source: LOVA projects’ final reports

	Harvest in t FW^a^	Reduced kg N	Reduced kg P	Reduced kg PO_4_-eq^b^
Mean	2232	11 651	865	7549
SD	2648	18 374	1194	11 144
Median	1300	4040	324	2793
Min	80	63	6	45
Max	10 418	69 000	4200	41 874
Sum	55 807	337 884	25 081	218 910
n	25	29	29	29
Sum for all 40 projects^c^	89 287	466 045	34 596	301 949

^a^FW = fresh weight. A conversion factor of 1 m^3^ = 1.5 t was used for projects which reported harvest in m^3^

^b^PO_4_ equivalents capture that both N and P reductions are needed for large parts of the Baltic Sea for mitigating eutrophication effects (Henryson et al. 2018), and that the general public values both N and P reductions (Ahtiainen et al. 2014b). The Redfield ratio suggests that 1 kg P is equal to about 7.2 kg N equivalents in terms of its potential to increase primary production (Guinée 2002; Henryson et al. 2018). The conversion to PO_4_-eq is consistent with the Redfield ratio, where 1 kg N is equal to 0.42 kg PO_4_-eq and 1 kg P is equal to 3.07 kg PO_4_-eq (GHK and BIOIS 2006)

^c^Some of the 40 projects did not report harvest and/or nutrient removal. Mean values of harvest and reduced kg N and P were assumed for these projects

The nutrient removal accomplished by the LOVA projects can be monetized using results from recent valuation studies on the benefits of reduced eutrophication effects in the Baltic Sea (Table 2). The relatively wide interval of USD_2018_ 17–73 kg^−1^ reduced PO_4_-eq is not surprising because of differences in valuation methods and geographical scope (Appendix S4). Using the mean value of USD_2018_ 38 kg^−1^ reduced PO_4_-eq captures numerical information from all these studies, implying that the nutrient removal accomplished by the LOVA projects from 2009 to 2018 corresponds to benefits amounting to USD_2018_ 11.5 million (USD_2018_ 38 kg^−1^ reduced PO_4_-eq × 301 949 kg reduced PO_4_-eq, Table 1). Beneficiaries are those whose wellbeing would increase due to reduced eutrophication effects that this nutrient removal contributes to realize, i.e., large parts of the general public in the Baltic Sea countries (Ahtiainen et al. 2014a).

**Table 2:** Benefit estimates kg^−1^ reduced PO_4_ equivalents computed based on results from recent valuation studies on reduced eutrophication effects in the Baltic Sea. See Appendix S4 for summaries of all studies and computation details

Study	Benefit kg^−1^ reduced PO_4_-eq in USD_2018_	Comment
Czajkowski et al. (2015)	17	Based on benefits for Sweden
Hasselström et al. (2020)	22	Based on the value of reducing N
Ahtiainen et al. (2014a)	30	Based on benefits for Sweden
Hasselström et al. (2020)	34	Based on the value of reducing P
Czajkowski et al. (2015)	40	Based on benefits for all Baltic Sea littoral countries
Nieminen et al. (2019)	43	Based on benefits for Finland
Östberg et al. (2012)	47	Based on benefits for a local coastal population in Sweden
Ahtiainen et al. (2014a)	73	Based on benefits for all Baltic Sea littoral countries

### Change of recreational opportunities on land

Beach-cast harvesting tends to increase the use of beaches by making them and the waters more physically accessible and also more pleasant to visit (Appendix S2b and LOVA project reports). Malodour caused by the release of sulphur compounds from beach-cast decomposition is perceived to decrease if beach-cast is gathered in piles, potentially because the malodour is concentrated around the piles (Appendix S2b). The value of the improved recreational opportunities is also indicated by the high degree of volunteering in harvest activities (cf. “Harvest costs” section) and by the willingness to pay for enhancing beaches for recreational activities in another case study in Sweden (Risén et al. 2017). Moreover, a recent model for monetizing recreational beach use indicates an addition of benefits when access to various activities increases (Pascoe 2019). The presence of machinery during harvesting of beach-cast can infer a temporary inconvenience and noise pollution, hence affecting recreational opportunities. However, most beach-cast is harvested during fall, winter and spring when beach-cast accumulates on the shores, although harvesting also occurs in the summer season for purpose of beach cleaning (Appendix S2c). Overall, a positive net benefit with respect to recreational opportunities for residents and tourists is therefore expected, but we refrain from monetizing it because of the lack of local valuation results on recreational beach use on Gotland.

### Habitat change on land

Harvesting affects the specific habitat associated to the bands of beach-cast and may result in decrease in species abundance and number of species. Some localities with beach-cast bands can be classified as a vulnerable habitat for threatened annual plant species according to the EU habitat directive (COM 1992). The adversity of the effects is correlated to the frequency and intensity of beach-cast removal (Zielinski et al. 2019), and moderate cleaning of beaches has been found to result in insignificant impacts on biodiversity and total animal biomass compared to untouched beaches (Malm et al. 2004). In contrast, eutrophication and massive macroalgae blooms may also lead to excessive biomass, which could reduce species abundance and number of species in marine communities (Green et al. 2014). This duality shows the difficulty in determining optimal levels of beach-cast in terms of habitat conservation. Furthermore, there is no set threshold at which the amount of beach-cast surpasses naturally occurring volumes. Consequently, defining a sustainable target for beach-cast harvesting is challenging. Work to develop protocols for sustainable beach-cast harvest is ongoing and includes leaving stretches of a beach untouched both spatially and temporally and leaving harvested beach-cast to decompose in situ to provide habitat functions (Zielinski et al. 2019). Such precautionary principles of harvest are applied to a number of beaches on Gotland, allowing only parts of the beaches to be cleaned and generally restricting harvesting to certain times of the year (Appendix S2c). While this is likely to reduce negative habitat effects, the net effect in terms of benefits or costs cannot be assessed from current data.

### Greenhouse gas emission change

Release of greenhouse gases from beach-cast harvesting may occur from the harvest activity itself, i.e., diesel combustion by harvesting machinery, but also from decomposition in beach-cast piles. Emissions of methane (CH_4_) and nitrous oxide (N_2_O) in anaerobic conditions and CO_2_ emissions in aerobic conditions both occur in bands and piles (Coupland et al. 2007; Han et al. 2014; Liu et al. 2019). Specific comparisons between anaerobic and aerobic conditions in piles and bands have, to our knowledge, not yet been studied. However, organic waste composting studies have identified important factors affecting organic decomposition and associated emissions, such as C/N-ratios of macroalgal composts (Amlinger et al. 2008), high temperature and humidity (which is correlated to distance from shoreline) increasing the release of both CH_4_ and N_2_O (Han et al. 2014; Sánchez et al. 2015; Liu et al. 2019) and also that pile size may affect anaerobic processes (Beck-Friis et al. 2000). Consequently, GHG emissions from beach-cast require further investigation to assess complexities of carbon flux rebound effects and mitigation protocols.

Given the knowledge gaps relating to GHG emissions from both beach-cast bands and piles, the present study only accounts for those GHG emissions which can be estimated, i.e., emissions of harvesting activities. The diesel consumption associated with machinery used for harvesting and transporting this machinery to and from harvest locations indicates CO_2_ emissions of about 3.3 kg t^−1^ FW (Appendix S5). Applying the social cost of carbon applied in transportation related CBAs in Sweden gives a total cost of USD_2018_ 0.24 million for the harvesting and associated transports of the LOVA projects (Appendix S5). This cost is incurred by the global population.

### Physical function change

Accumulating bands of beach-cast over the course of years can alter the geomorphology and topography of a beach and potentially provide natural coastal protection by supporting dune formation and providing sand stabilization (De Falco et al. 2008; Mossbauer et al. 2012; Milledge and Harvey 2016). Consequently, beach-cast harvesting could reduce such protection and cause shoreline erosion. This could be exacerbated if substantial amounts of sand are removed with the beach-cast and by compression of sand layers by heavy machinery (De Falco et al. 2008; Milledge and Harvey 2016). However, the severity of beach erosion may differ between sites depending on, e.g., topography and substrate (Mörner and Finkl 2019). On Gotland, an estimated 30% of sand beaches possess characteristics that imply risks for future erosion (SGU 2020). The importance of beach-cast for reducing erosion should be evaluated in such areas, and harvesting on such sites should be limited as it may entail future costs of restoration. The future nature of this potential cost and the lack of risk quantifications refrain us from monetization.

### Harvest costs

Harvesting beach-cast involves the use of machinery such as tractors suitable for harvest and piling, and associated labour. The LOVA projects typically hired this type of harvest service from local businesses and their fees are assumed to cover not only the operational costs for the harvests (e.g., fuel and labour) but also investment and maintenance costs for the machinery. The total invoiced costs for all projects amounted to about USD_2018_ 0.62 million (Table 3). The projects also reported opportunity costs of time for unpaid work in terms of volunteering; such work includes harvesting as well as project planning and administration. The total harvest costs including volunteering were about USD_2018_ 1.1 million (Table 3). These costs are partly incurred by the Swedish government through the LOVA grant system and partly by the organizations and individuals involved in the LOVA projects.

**Table 3:** Harvest costs for the LOVA projects (2009–2018) in USD_2018_. (See Appendix S6 for costs expressed in SEK_2018_)

	Invoiced costs^a^	Volunteering through harvest vehicle driving^b^	Other volunteering^b^	Total costs
Mean	15 525	3731	9158	28 414
SD	20 039	10 653	22 540	28 372
Median	9991	0	5304	21 724
Min	882	0	0	1479
Max	108 431	46 997	60 745	110 732
Sum for 38 projects^c^	589 949	141 777	348 011	1 079 737
Sum for all 40 projects^d^	620 999	149 239	366 328	1 136 566
Cost t^−1^ FW harvest^e^	6.9	1.7	4.1	13
Cost kg^−1^ reduced PO_4_-eq^f^	2.1	0.49	1.27	3.8

^a^Invoiced costs do not include 25% VAT because it is assumed that harvesting activities do not displace other types of production, cf. Johansson and Kriström (2018)

^b^For volunteering, Swedish authorities apply a standard value of SEK 500 h^−1^ (USD 58) for drivers of tractors and other vehicles, and SEK 200 h^−1^ (USD 23) for all other activities

^c^Cost data were reported for 38 of 40 projects

^d^Mean costs were assumed for 2 projects with missing cost data

^e^Based on a total harvest of 89 287 t FW, see Table 1. Cost variability across individual projects is illustrated in Appendix S6

^f^Based on a total nutrient removal of 301 949 kg PO_4_-eq, see Table 1. Applying nutrient concentration results from Franzén et al. (2019) give similar average costs (Appendix S6)

### Transaction costs

Transaction costs related to the LOVA project system are delimited to the costs for administrating LOVA applications and reporting at CABG. Administrative costs incurred by LOVA project owners and other project participants are probably to a large extent included in the volunteering reported in harvest costs. Based on annual reporting from CABG to the Swedish Agency for Marine and Water Management, the total administrative costs incurred by the Swedish government are estimated to about USD_2018_ 0.39 million for the period of 2009–2018 (Appendix S7).

### Other consequences

Other consequences of relevance for a CBA include other types of air emissions than GHGs from harvest machinery as well as less tangible aspects such as the impact on social cohesion through the local collaborative efforts that the LOVA projects have entailed. The former can be monetized by applying social costs of NO_x_ and NH_3_ emissions applied in transportation related CBAs in Sweden, and amount to about USD_2018_ 0.0018 million (Appendix S5). The latter is difficult to monetize, but would add to society’s social capital, and interview data indicate a positive impact of the increased community networking implied by the LOVA projects (Appendix S2d). The LOVA projects have also supported knowledge-building about harvest practices that might imply cost reductions in future projects. There might also be marine ecosystem effects beyond those associated with nutrient removal; for example, indications of improved conditions for flatfish recruitment because of less humus accumulation on coastal sea-beds in initial studies should be subject to further investigation (Martinsson 2015).

### Sensitivity analysis and conclusions about economic profitability

Based on benefits and costs that can currently be monetized, the total benefits of the beach-cast harvest in the LOVA projects (USD_2018_ 11.5 million) exceed the total costs (USD_2018_ 1.73 million) (Table 4). This would be the case also if the lower end of the interval of USD_2018_ 17–73 kg^−1^ reduced PO_4_-eq is applied for the benefits of nutrient removal instead of the mean value of USD_2018_ 38 kg^−1^ reduced PO_4_-eq: 17 × 301 949 kg reduced PO_4_-eq = USD_2018_ 5.1 million. Break-even would require a benefit of nutrient removal amounting to USD_2018_ 5.7 kg^−1^ reduced PO_4_-eq, i.e., a figure almost three times lower than the lower end of the interval of USD_2018_ 17–73 kg^−1^ reduced PO_4_-eq and about 6.7 times lower than the mean value of USD_2018_ 38 kg^−1^ reduced PO_4_-eq. Given this mean value, break-even occurs alternatively at a nutrient removal amounting to 45 526 kg PO_4_-eq, which is again about 6.7 times lower than the removal reported by the LOVA projects. Break-even would thus require that benefits per kg reduced PO_4_-eq and/or the nutrient removal in kg PO_4_-eq are substantially overestimated, other things being equal. While this cannot be precluded, it should be recalled that the benefits of nutrient removal are based on several recent and peer-reviewed valuation studies (Appendix S4) and that an alternative estimation of total nutrient removal did not result in any lower estimates (Appendix S3). As to the cost side, the harvest costs of USD_2018_ 1.1 million have to increase by a factor of ten before reaching break-even, other things being equal. A comparison with other harvest cost estimates suggests that such a substantial underestimation of harvest costs is not likely (Appendix S6). Further, the LOVA projects have small incentives to underreport harvest costs, because grants through the LOVA grant system are based on the projects’ reported costs. We therefore conclude that a positive economic profitability is the most probable outcome of this CBA. It should be acknowledged that the non-monetized consequences add uncertainty to this conclusion, but the non-monetized costs would have to be remarkably substantial to outweigh the monetized net benefit and the non-monetized benefit due to improved recreational opportunities on beaches subject to beach-cast harvest.

**Table 4:** Summary of costs and benefits associated with the beach-cast harvest in the LOVA projects, 2009–2018

Type of consequence	Benefit, million USD_2018_	Cost, million USD_2018_
Nutrient removal from the marine system	11.5	
Change of recreational opportunities on land	Net effect likely to be a clear benefit	
Habitat change on land	Net effect inconclusive, but probably small	
GHG emission change from bands to piles	Net effect difficult to ascertain	
GHG emissions from harvest machinery and associated transports		0.24
Physical function change		A potential cost in the future
Harvest costs		1.1
Transaction costs		0.39
Other consequences	Net effect difficult to ascertain	

## Discussion

We found that the harvest activities in the analyzed LOVA projects are likely to have been economically profitable to society, though there are considerable uncertainties that should be acknowledged and deserve further investigations, including currently non-monetized benefits and costs. For example, the benefits of nutrient removal and improved recreational opportunities could be monetized more precisely by carrying out local valuation studies that are designed specifically for Gotland. Similarly, more detailed protocols for reporting harvesting costs, nutrient concentrations in beach-cast and harvested beach-cast fate, combined with systematic follow-up studies, could also reduce uncertainties. Another uncertainty is due to potential nutrient run-off to the sea from piles of harvested beach-cast. If piles are left unused at unsuitable places from a run-off perspective, the nutrient removal benefits in the CBA become temporary, but could be secured by identifying piling locations that minimize run-off risks and/or using beach-cast in a way that recirculates nutrients (see below). Note also that the benefits related to improved recreational opportunities on land arise largely from the mere relocation of beach-cast from beaches to piles.

The positive economic profitability suggests that policies that helped realizing the analyzed LOVA projects have been motivated, particularly because nutrient removal can be viewed as a positive externality of harvesting. The policy instrument was in this case a Swedish national grant system, which suggests that the regional approach to standing applied in the CBA should be discussed. It could be argued that a Swedish grant system is consistent with giving standing to only Swedish citizens in a CBA. Such a national approach to standing would motivate a delimitation of nutrient removal benefits to those enjoyed by Swedes, i.e., the estimates of USD_2018_ 17, 30 and 47 kg^−1^ reduced PO_4_-eq in Table 2. However, even the lowest of these three estimates still suggests a positive economic profitability. That is, also applying a national approach to standing in the CBA gives support to the existence of a national policy such as the LOVA grant system. Additionally, economic principles for policy development suggest that biomass harvesting activities with low current market viability in terms of biomass value but significant nutrient uptake potential, are particularly suitable for including in taxpayer funded payment schemes for nutrient uptake (Hasselström and Gröndahl 2021).

As to future beach-cast harvesting, continued subsidization is an option, which suggests that the role of beach-cast harvest in relation to other subsidized land- and sea-based nutrient removal measures should be explored further, i.e., adding beach-cast harvest into cost-effectiveness analyses such as Hyytiäinen et al. (2015). Such an analysis should consider that different measures might have very different side-effects with respect to, for example, recreation and habitat conditions (Jensen et al. 2019) and involve different transaction costs (McCann 2013). The significance of the latter is illustrated by the fact that this accounted for 23% of the total monetized costs in the LOVA project case (Table 4).

Also the choice of system boundary is crucial: The value chain steps in the present study focus on harvesting, but extending the analysis to post-harvest processes might affect the economic outcome. On Gotland, there is small-scale agricultural use of beach-cast that may increase soil fertility and to some extent substitute mineral fertilizer and thus possibly imply cost reductions for farmers (Nabti et al. 2017). However, the content of heavy metals in beach-cast adds some uncertainty concerning its use in agriculture and calls for more research on the variations of heavy metal concentrations in beach-cast and uptake in different agricultural plants (Franzén et al. 2019). Other potential types of use include the production of biogas (Blidberg et al. 2012; Risén et al. 2017), biochar (Macreadie et al. 2017) and insulation (Widera 2014), see also Chubarenko et al. (2021). A wider system boundary would also motivate the accounting of carbon flows and net GHG reduction potential resulting from different use options, which might give additional motivation for funding beach-cast management or give direct financial income through carbon trading. In-depth assessments of post-harvest GHG emissions from beach-cast storage and use, and of various use options and their financial viability are key aspects to explore to optimize future beach-cast management.

Such assessments should also consider a reasonable scale of beach-cast harvest. On Gotland, it has been suggested that the length of suitable beaches to harvest are 3–5 times larger than those currently being harvested (Ulf Smedberg, pers. comm.). Broadening the geographical perspective nationally and internationally implies considerable harvest opportunities. For example, the total beach-cast biomass potential for the whole southern Sweden can be estimated to 241 000–257 000 t FW yr^−1^ (Blidberg et al. 2012), of which only a tiny proportion is being harvested. However, increasing harvests could have a non-linear impact on harvest costs and other consequences, for example, affecting places where bands of beach-cast are relatively more important as a habitat or provider of physical functions. This suggests a need to identify criteria to support the selection of suitable sites for harvest, to develop cost-effective harvest protocols well-adapted to minimize negative impacts on shoreline stability and ecosystems, and to take into account that inaction motivated by precaution with respect to particular habitats might to some extent be undesirable from a broader systems perspective.

The case of Gotland illustrates synergies—between the local interest for improved areas for beach recreation and nutrient removal from the sea—that can stimulate the kind of collaborative stewardship across landscapes and seascapes that is viewed as necessary in fostering sustainability (Folke et al. 2021). Funding predictability and consistency are needed to ensure long-term harvesting and sustain the attained collaboration among local actors, but national subsidy systems entail some uncertainty because priorities in national environmental policy may change. The solution may be a system where those who benefit the most from beach-cast harvest also contribute financially to the system. One such group is tourists visiting Gotland. While Sweden’s Right of Public Access implies that tourists can visit beaches without any entrance fee, a funding system involving an environmental fee upon arrival to Gotland is an option that could be investigated. The total monetized costs of USD_2018_ 1.73 million (Table 4) could be covered by a fee of USD 0.79 per passenger to and from Gotland during one year (Fig. 1). Funding could also be bolstered by sustainable use of beach-cast. Overall, the great potential in connecting marine, coastal and terrestrial social-ecological systems should be subject to further research.

## Conclusions

This CBA suggests that the analyzed beach-cast harvesting on Gotland during 2009–2018 has been economically profitable to society, not least because of removal of 466 t N and 35 t P from the heavily eutrophicated Baltic Sea (Table 1), though it should be assessed in detail how the removal is best secured through post-harvest practices that minimizes potential nutrient run-off. Beach-cast harvest could thus potentially play an economically motivated role in nutrient management, and further investigations of benefits and costs for different scales of harvests are warranted. For example, local valuation studies can illuminate the extent of benefits considerably; this is not least true in an international perspective, where varying coastal livelihood situations may entail different benefits. Further, collection of detailed harvest data that can reveal crucial organizational, technical and other determinants of cost variability would help identify cost-effective harvest practices that can support sustainable harvest methods with minimal ecological disturbance. Beach-cast use options should also be brought into the picture, including the potential to contribute to reduced GHGs release. Moreover, the organizational, technical, legal and financial lessons learnt from the considerable experience on Gotland should be compiled and utilized for developing concrete guidelines on where and how harvest can be made in a way that minimizes potentially negative impact on ecosystems and shoreline stability, paying due attention to local conditions but also to what can be generalized to a wider national and international context.

## Supplementary Information

Below is the link to the electronic supplementary material.

Supplementary file1 (PDF 354 kb)
