# Supplementary material for: Cost–benefit analysis of beach-cast harvest: Closing land-marine nutrient loops in the Baltic Sea region
Source: Ambio. 2021 Nov 17;51(5):1302–13. doi: 10.1007/s13280-021-01641-8 (PMC8931131; doi:10.1007/s13280-021-01641-8)
Supplement: Supplementary file 1 — Supplementary file1 (PDF 354 kb) [file 13280_2021_1641_MOESM1_ESM.pdf]

Supplementary Material

Cost-benefit analysis of beach-cast harvest: Closing land-marine nutrient loops in the Baltic Sea region

Tore Söderqvist[a], Hanna Nathaniel[b], Daniel Franzén[b], Frida Franzén[c], Linus Hasselström[b], Fredrik Gröndahl[b], Rajib Sinha[b], Johanna Stadmark[d], Åsa Strand[e], Ida Ingmansson[c], Sofia Lingegård[b], Jean-Baptiste Thomas[b]*

[a] Holmboe & Skarp AB, Norr Källstavägen 9, SE-148 96 Sorunda, Sweden

[b] KTH Royal Institute of Technology, Department of Sustainable Development, Environmental Science and Engineering, Teknikringen 10B, SE-100 44 Stockholm, Sweden

[c] Tyréns AB, Peter Myndes Backe 16, SE-118 46 Stockholm, Sweden

[d] IVL Swedish Environmental Research Institute, Box 53021, SE-400 14 Göteborg, Sweden

[e] IVL Swedish Environmental Research Institute, Kristineberg 566, SE-451 78 Fiskebäckskil, Sweden

* Corresponding author. E-mail: jean-baptiste.thomas@abe.kth.se.

S1. Summary of earlier literature on CBA of beach-cast harvest

Yang et al. (2018) presented an overview of the costs associated with green tides in the Chinese Yellow Sea. In this setting, damage costs include damage on tourism, recreation and aquaculture, as well as management costs related to harvesting and the protection of aquaculture. They showed that preventing blooms by lowering nutrient load from local aquaculture is more profitable to society than mitigating the consequences of blooms by harvesting and protective measures. The prevention of eutrophication has been shown to be economically profitable also in the Baltic Sea (Gren et al. 1997; Hyytiäinen et al. 2015; Scharin et al. 2016). However, still remaining is the more niched question on whether the benefits of beach-cast harvest as a specific eutrophication mitigation measure is an economically profitable venture, taking into account the costs of harvesting and the benefits of, for example, nutrient uptake and improved recreational opportunities. Concerning this more specific issue, Gisselman (2014) applied CBA for assessing the use of macro algae and reed from the bay of Burgsviken in Southern Gotland for biogas production. The monetized benefits and costs gave a social loss, but all benefits were not monetized and it was judged that a more complete monetization could result in a social gain. In contrast to the present study, the macro algae collection concerned free-floating macro algae, and no data on nutrient contents of collected algae were used; Gisselman (2014) cautiously regarded his study as a pre-study. Risén et al. (2017) applied the contingent valuation method for estimating non-market values of harvesting beach-cast for the purpose of removing nutrients from the Baltic Sea and producing biogas and biofertilizer in the case of Trelleborg Municipality in Southern Sweden, but did not analyze costs of such harvest and production. An overview by Blidberg et al. (2012) included such costs, based on the case of Trelleborg, and the benefits of nutrient uptake and indicated a net gain for society. However, this overview was not based on a strict CBA framework, which implies risks of overlapping benefit and cost items. Ofori and

Rouleau (2020) conducted a willingness to pay study in Elmina, Ghana, for periodically removing *Sargassum* seaweeds from beaches. Mossbauer et al. (2012) gave examples of costs of beach-cast management for the case of the German Baltic Sea coast and also the potential commercial value of beach-cast, but did not include a full CBA. Charlier et al. (2007) presented cost estimates of beach-cast harvesting in Bretagne, France. Mossone et al. (2019) studied preferences of beachgoers for beaches with or without beach-cast at a number of Mediterranean seaside resorts, but not in a eutrophication context or with including costs of removing beach-cast. The benefits and costs of beach cleaning have been studied (e.g., Zielinski et al. 2019; Cruz et al. 2020; Mouat et al. 2010), but such cleaning is often about collecting litter and not related to eutrophication mitigation.

S2. Information from interview study with stakeholders

S2a (referred to in Section 3). Complementary data for the cost-benefit analysis was obtained from another research project conducting a stakeholder analysis of the beach-cast system on Gotland and applying the same system boundaries and scope. In this project, semi-structured interviews were carried out in December 2019–May 2020 with 18 stakeholders of Gotland-based beach-cast harvesting activities, representing six non-governmental organizations (NGOs), six actors in the public sector and six actors in the private sector. The interviews provided stakeholder insights to the practical aspects and functioning of the beach-cast system, which served as a knowledge base that could be used for shedding light on consequences studied in the CBA. The interviewees were selected through snowball sampling to represent all scales of operation within the beach-cast system and grouped accordingly into NGOs, the public sector and the private sector. The interview results were scanned for

statements relating to ‘ecosystem service provision’ and these findings are referred to in this study, relating the statements to respective stakeholder groups.

S2b (referred to in Section 4.2). Stakeholders from all three groups, *NGOs*, *public* and *private sector*, emphasized the positive effects on recreational opportunities from beach-cast removal, both on land and at sea. They perceived the collection of beach-cast in piles as making the water more accessible, as well as being a way of concentrating the inevitable malodour to certain spots, a conduct that they viewed as increasing people’s pleasure of visiting the beaches.

S2c (referred to in Sections 4.2 and 4.3). All stakeholder groups, *NGOs*, *public* and *private sector*, stated that beach-cast harvesting is primarily limited to certain beaches and periods of the year, foremost fall, winter or spring. The public sector claimed that the reason for this is the uncertainties regarding the habitat effects, wherefore a precautionary stance is applied. NGOs and the private sector, however, believed or speculated that the reason for this procedure is administrative restrictions without tying them to concerns for habitats.

S2d (referred to in Section 4.8). Stakeholders perceived local networking and collaborations between and among actors as a positive consequence of the LOVA system. All stakeholder groups expressed the emergence of this social cohesion, which was a reoccurring topic throughout the interviews, explicitly stated or embedded in the responses.

S3. Nutrient removal from the marine system

The nutrient removal reported by the LOVA projects are based on mandatory chemical analyses of beach-bast carried out by professional laboratories. The reported removal can still be expected to be uncertain, because there is no detailed information available about how beach-cast samples were taken and a procedural variability can be expected among the

projects. We therefore also make use of scientifically published results on N and P content in beach-cast from 15 sites situated in the southernmost part of Gotland (Franzén et al. 2019). While 15 sites are a small sample, a consistent protocol for sampling and chemical analysis were followed and indicated a mean concentration of 5.42 kg N t^{-1} FW and 0.55 kg P t^{-1} FW (i.e., 3.96 kg PO_4-eq t^{-1} FW) (Franzén et al. 2019, Table 1). Applying these estimates on the total harvest of the LOVA projects (about 90 000 t FW) results in a somewhat higher nutrient removal than that reported by the projects: 487 800 kg N and 49 500 kg P. This indicates that the LOVA projects have not overestimated the nutrient removal.

S4. Benefits of nutrient removal from the marine system

The benefits of reduced eutrophication effects in the Baltic Sea are relatively well-studied thanks to a number of valuation studies from early 1990's onwards. We have chosen to focus on four valuation studies published in peer-reviewed scientific journals during the last ten years (after 2010) in order to have as recent and scientifically reliable results as possible on people's willingness to pay:

- Ahtiainen et al. (2014) implemented a contingent valuation survey in 2011 through questionnaires to households in the nine littoral countries of the Baltic Sea. The questionnaire included a description of the Baltic Sea and its environmental status, questions about leisure activities at the sea and a valuation scenario including questions about willingness to pay (WTP). The total WTP for all nine countries for reaching the eutrophication objectives of the Baltic Sea Action Plan (BSAP) was estimated to €$_{2011}$ 3603 million yr^{-1} (ibid., Table 7, p. 21). The corresponding total WTP among Swedes was found to be €$_{2011}$ 572.7 million yr^{-1} (ibid., Table 7, p. 21).

- Czajkowski et al. (2015) studied recreational values associated with the Baltic Sea by using the travel cost method. Data were obtained from a questionnaire to households in the nine littoral countries of the Baltic Sea. The annual benefits of recreation enjoyed at an improved environmental status of the sea was compared to those enjoyed at current status. The change in total consumer surplus for all nine countries because of a reduced eutrophication was estimated to $€_{2011}$ 1969 million yr^{-1} (ibid., Table 3, p. 214). The corresponding estimate for Sweden was $€_{2011}$ 336.1 million yr^{-1} (ibid., Table 3, p. 214).
- Nieminen et al. (2019) used contingent valuation for estimating the WTP in Finland for achieving Good Environmental Status (GES) in Finnish marine waters. GES is the aim of the EU Marine Strategy Framework Directive. The total WTP among households in Finland for achieving GES was estimated to $€_{2017}$ 432–509 million yr^{-1} (ibid., Table 4, p. 186), i.e., on average $€_{2017}$ 470.5 million yr^{-1}.
- Östberg et al. (2012) applied contingent valuation for valuing a water quality improvement in two coastal areas in Sweden, one at the Skagerrak part of the Swedish Westcoast and one in the SW part of the Stockholm Archipelago, which is a part of the Baltic Sea. The valued improvement corresponded approximately to achieving Good Ecological Status with respect to eutrophication, as defined by the EU Water Framework Directive. For the Baltic coastal area, a midpoint estimate of mean monthly WTP per household was SEK_{2009} 102.06 (ibid., Table 5, p. 173), i.e., about $€_{2009}$ 10.2. Kinell et al. (2012, pp. 16-17) estimated the local population being sampled for the survey to 52 308 households, which implies a total WTP amounting to $€_{2009}$ 6.4 million yr^{-1} (10.2 × 52 308 × 12).

Linking the results of the valuation studies to amounts of nutrient removal is a challenge because the studies do not specify what nutrient reductions are necessary to accomplish the

reduced eutrophication effects subject to valuation. However, the situation is relatively straightforward for Ahtiainen et al. (2014) and Czajkowski et al. (2015), because these studies valued reduced eutrophication effects for the whole Baltic Sea and reduction objectives have been defined in BSAP for the sea as a whole, see more below. While there is some uncertainty concerning whether these objectives are sufficient for achieving the improved environmental status that BSAP is aiming at, the complexity increases in the case of the valuation studies focusing on particular parts of the Baltic Sea: Nieminen et al. (2019) and Östberg et al. (2012). This is because eutrophication effects in these sea areas might be dependent not only on the local nutrient load but also on nutrient loads from other parts of the sea.

The Helsinki Commission (HELCOM 2015, p. 98) estimated that the annual load of nitrogen and phosphorus to the Baltic Sea must be reduced by 70 988 t N and 12 132 t P in comparison to a baseline period of 2008–2010 for achieving BSAP objectives by 2021, i.e., an annual load reduction of 67 060 t PO_4-eq (70 988 × 0.42 + 12 132 × 3.07). We therefore interpret these reduction figures as the necessary annual reduction required for achieving the improvement that was valued in Ahtiainen et al. (2014) and Czajkowski et al. (2015) and for which estimates of annual total benefits are thus available. The results of Ahtiainen et al. (2014) thus suggest a benefit of $€_{2011}$ 54 kg^{-1} reduced PO_4-eq ($€_{2011}$ 3 603 000 000 yr^{-1} divided by 67 060 000 kg PO_4-eq yr^{-1}), whereas the corresponding benefit based on Czajkowski et al. (2015) is $€_{2011}$ 29 kg^{-1} reduced PO_4-eq ($€_{2011}$ 1 969 000 000 yr^{-1} divided by 67 060 000 kg PO_4-eq yr^{-1}). The latter estimate thus constitutes 54 % of the former one, and one reason for this is likely to be methodological: Czajkowski et al. (2015) was a travel cost study able at estimating recreational benefits only, whereas the contingent valuation method used by Ahtiainen et al. (2014) can estimate total benefits in the sense that the method can capture both use and non-use values.

We now proceed by comparing the results above to those of Nieminen et al. (2019) and Östberg et al. (2012). As mentioned above, these two studies were about particular parts of the Baltic Sea, which means that the task of relating their valuation results to nutrient reductions becomes more challenging. We approach this task in the following way.

For Nieminen et al. (2019), there is a need to relate to nutrient reductions necessary for Finnish marine waters to reach GES. This should not be confused with nutrient reduction targets for Finland, because nutrients reach Finnish waters also from other parts of the Baltic Sea. We therefore take the nutrient reduction targets for the Baltic Sea mentioned above as a point of departure and assume that reasonable targets for Finnish waters is proportional to the area of Finnish exclusive economic zone (81 553 km^2, Marineregions.org 2020a) in relation to the area of the whole Baltic Sea (415 266 km^2, HELCOM 2006, p. 6), i.e. 19.6 %. This implies an annual reduction for Finnish marine waters amounting to 0.196 × 67 060 = 13 144 t PO_4-eq. Relating the average estimate of total benefits in Nieminen et al. (2019) to this target results in $€_{2017}$ 36 kg^{-1} reduced PO_4-eq ($€_{2017}$ 470 500 000 yr^{-1} divided by 13 144 000 kg PO_4-eq yr^{-1}). It should be noted that relating the estimated benefits in Nieminen et al. (2019) to reduced eutrophication only implies a risk for overestimating the benefits of reduced eutrophication, because achieving GES is also about improving other environmental aspects. On the other hand, Nieminen et al. (2019) reported that reduced eutrophication effects were prioritized by survey respondents together with reducing toxic substances.

Applying a corresponding proportional approach to Swedish waters for the total benefits among Swedes in Ahtiainen et al. (2014) and Czajkowski et al. (2015) implies the following: The area of the Swedish exclusive economic zone (148 523 km^2; Marineregions.org 2020b; VISS 2020)[1] constitute 35.8 % of the area of the whole Baltic Sea, which corresponds to an annual reduction to 24 007 t PO_4-eq. For Ahtiainen et al. (2014), € 572 700 000 yr^{-1} is divided

[1] Swedish total EEZ: 155 347 km^2. Skagerrak part of Swedish total EEZ: 6824 km^2.

with 24 007 000 kg PO_4-eq yr^{-1}, which gives €$_{2011}$ 24 kg^{-1} reduced PO_4-eq, and for Czajkowski et al. (2015), the corresponding figure is €$_{2011}$ 14 kg^{-1} reduced PO_4-eq (€$_{2011}$ 336 100 000 yr^{-1} divided by 24 007 000 kg PO_4-eq yr^{-1}).

Östberg et al. (2012) estimated benefits for improved water quality through reduced eutrophication effects in a bay area in SW part of the Stockholm Archipelago: The bay of Himmerfjärden with adjacent minor bays. Larsson et al. (2011, Table 3.1) estimated the total nutrient load to the area to 912 t N and 41 t P in 2010. Based on official nutrient reduction targets for Himmerfjärden[2], we approximate the necessary annual nutrient reductions to 273.6 t N and 20.5 t P, i.e., 177.8 t PO_4-eq (273.6 × 0.42 + 20.5 × 3.07). This suggests a benefit amounting to €$_{2009}$ 36 kg^{-1} reduced PO_4-eq (€$_{2009}$ 6 400 000 yr^{-1} divided by 177 800 kg PO_4-eq yr^{-1}).

As an additional comparison, Hasselström et al. (2020) reported midpoint estimates of €$_{2017}$ 7.6 kg^{-1} reduced N and €$_{2017}$ 86.5 kg^{-1} reduced P. Applying PO_4 equivalents, 1 kg reduced N corresponds to 0.42 kg reduced PO_4-eq, which suggests a benefit of €$_{2017}$ 18 kg^{-1} reduced PO_4-eq (7.6 divided by 0.42). Analogously, 1 kg reduced P corresponds to 3.07 kg reduced PO_4-eq, which suggests a benefit of €$_{2017}$ 28 kg^{-1} reduced PO_4-eq (86.5 divided by 3.07).

Table S1 summarizes all results with respect to benefits per reduced kg PO_4-eq, including conversion to €$_{2018}$ and USD$_{2018}$. The relatively wide range of USD$_{2018}$ 17–73 kg^{-1} reduced PO_4-eq (mean: 38) is not surprising, given the variation in valuation approaches, geographical scope and the challenge of associating benefits with necessary nutrient reductions. We also assume that average benefit estimates are applicable to beach-cast harvest, which suggest a situation where beach-cast harvest is considered as integrated into an assembly of many different measures for combatting eutrophication in the Baltic Sea. A considerably more

[2] About 50 % P reduction and 30 % N reduction, see https://viss.lansstyrelsen.se/Waters.aspx?waterMSCD=WA55952587 (accessed 21 March 2019).

refined analysis, such as applying meta-analysis function benefit transfer (e.g., see Johnston et al. 2021), is left here as a suggestion for future research. Finally, it should be observed that the estimates of Hasselström et al. (2020) are partly based on older valuation studies than the other ones in Table S1 and might therefore be less relevant. However, excluding those estimates does not affect the range of USD_{2018} 17-73 kg^{-1} reduced PO_4-eq, and the influence on the mean value is limited to a 10 per cent increase from USD_{2018} 38 to 42.

Table S1. Benefit estimates kg^{-1} reduced PO_4 equivalents from valuation studies.

Study	Benefit kg^{-1} reduced PO_4-eq			Comment
	Original midpoint estimate	Estimate in $€_{2018}$[a]	Estimate in USD_{2018}[b]	
Czajkowski et al. (2015), based on benefits for Sweden	$€_{2011}$ 14	15	17	Travel cost study, i.e., recreational benefits only. Necessary nutrient reduction is assumed to be proportional to Sweden's marine area with the total Baltic Sea area.
Hasselström et al. (2020), derived from the value of reducing N	$€_{2017}$ 18	18	22	Based on several valuation studies, partly relatively old ones (earlier than 2010).
Ahtiainen et al. (2014), based on benefits for Sweden	$€_{2011}$ 24	25	30	Contingent valuation study, i.e., total benefits are captured. Necessary nutrient reduction is assumed to be proportional to Sweden's marine area with the total Baltic Sea area.
Hasselström et al. (2020), derived from the value of reducing P	$€_{2017}$ 28	29	34	Based on several valuation studies, partly relatively old ones (earlier than 2010).
Czajkowski et al. (2015), based on benefits for all Baltic Sea countries	$€_{2011}$ 29	34	40	Travel cost study, i.e., recreational benefits only. Benefits are related to the necessary nutrient reduction for the whole Baltic Sea.
Nieminen et al. (2019), based on benefits for Finland	$€_{2017}$ 36	36	43	Contingent valuation study, i.e., total benefits are captured. Captures Good Environmental Status as a whole, i.e., not only reduced eutrophication effects. Necessary nutrient reduction is assumed to be proportional to Finland's marine area with the total Baltic Sea area.
Östberg et al. (2012), based on benefits for a local coastal population in Sweden	$€_{2009}$ 36	40	47	Contingent valuation study, i.e., total benefits are captured.
Ahtiainen et al. (2014), based on benefits for all Baltic Sea countries	$€_{2011}$ 54	61	73	Contingent valuation study, i.e., total benefits are captured. Benefits are related to the necessary nutrient reduction for the whole Baltic Sea.

[a] Original estimates were adjusted for inflation by using consumer price index (OECD 2021; World Bank 2021). For the studies involving several Baltic Sea countries, the adjustment was based on the mean inflation rate in these countries.
[b] The $€_{2018}$ figures were converted to USD_{2018} by using the mean USD/€ currency rate for 2018, i.e. 1.1810 (ECB 2021).

S5. Emission estimates for the harvesting of beach-cast on Gotland

This section details the process of estimating emissions (combustion emissions of CO_2, NO_x, NH_3, plus aggregated climate impacts last taking a life cycle perspective) resulting from typical beach-cast harvesting activities on Gotland. The first part of the section presents the case that forms the basis of the estimations. Thereafter, emissions data extracted from the Ecoinvent database (v.3.3) and USLCI database are presented and used to estimate average emissions per day. Ecoinvent and USLCI are two of the most established databases that provide documented process data and associated emission/impact factors used in environmental assessments such as life cycle assessment (LCA). Each database is compiled using different methods, data sources, and geographical contexts, and as a result, emission/impact factors for processes often vary to some degree. They are used side-by-side in the present calculations for reference, because they both contained suitable processes for diesel combustion.

The amount and types of emissions resulting from the combustion of diesel depends to a large extent on the type of combustion engine being used (i.e., which are subject to varying degrees of efficiency, filtration, etc.) and on how one delimits the system boundaries (e.g., end-of-pipe emissions from the motor’s exhaust vs. full life cycle perspective) and functional unit of the study (e.g., emissions resulting from 1 km of transport in city vs. 1 km of transport on motorway). The emissions calculations hereafter presented should not be referred to as accurate modelled predictions nor as measured emissions data, but rather, these estimates should be considered as indicative of potential emissions from beach-cast harvesting activities.

Case summary. The case information (i.e., harvesting process description, fuel consumption, transport estimates, biomass yield estimates, etc.) is based on data received by Linus Hasselström and Jean-Baptiste Thomas during an interview carried out with Ulf Smedberg of

Smedbergsgård AB, in December 2018. Ulf Smedberg is a major actor on Gotland with respect to carrying out beach-cast harvesting and his services were employed in many of the LOVA projects. Key information necessary for the calculations includes:

(i) Approximately 30 km of beaches cleaned per year by Ulf Smedberg, perhaps totaling between 10k tons and 12k tons fresh weight of harvested biomass yr^{-1}.
(ii) Total travel is usually around 150–200 km day^{-1} consuming around 20 L diesel per hour, travelling at 20–65 km hr^{-1} (circa 50 km hr^{-1} on average)
(iii) 10-15 hrs of harvest time per day, diesel consumption around 8–10 L (9 L on average) hr^{-1} when at work (during harvest), harvest around 300–400 tons on a good day, usually between 100–300 tons, conservative estimate would be circa 150 tons day^{-1}.

From this information one can estimate average diesel consumption:

≈70 L for transport to and from the harvest location

≈113 L for harvesting

Total ≈ 183 L day^{-1} (i.e., for around 150 tons of harvested beach-cast)

Emissions data for combusted diesel. The emissions from two alternative diesel combustion processes were identified using SimaPro (V8.5). SimaPro is one of the commonly utilised LCA modelling tools. For each of these processes, emissions of CO_2, NO_X and NH_3 resulting only from the combustion of diesel were exported. In addition, the climate impacts (GWP100, expressed in kgs of CO_2 equivalents) for the full life cycle of each of these processes were calculated using the CML 2 Baseline 2000 (V2.05) method, for reference.

The first process, "Diesel, burned in agricultural machinery {GLO}| diesel, burned in agricultural machinery | Alloc Def, U", was selected from the Ecoinvent database (V3.3). In addition to the diesel's production, distribution, storage and combustion, the dataset includes contributions for heavy road transport with tractor, an agricultural trailer, tyres, agricultural shed for the vehicles when not in use. The unit for this process is 1 MJ.

The second process, “Diesel, combusted in industrial equipment/US”, was selected from the USLCI database. This dataset was selected to provide additional perspective specifically on diesel combustion, and is described as representing diesel combustion in industrial applications, such as mobile refrigerator units, generators, pumps and similar equipment. The unit for this process is 1 L of diesel.

Given the different units used for each of these processes, the first was also converted* to 1 L by multiplying by (1/0.026).

**Conversion factors from MJ to kg to L for common diesel oil*

1	MJ	0.022	kg	0.026	L
38	MJ	0.83	kg	1	L
45	MJ	1	kg	1.2	L

Estimated emissions from case study. The estimated emissions resulting from the harvesting of 150 tons of beach-cast are presented below and expressed both per 150 ton harvest (total) and per ton of harvested beach-cast (i.e., total divided by 150), following practices undertaken at Smedbergsgård AB. These are calculated using the emissions extracted from both the Ecoinvent process and the USLCI process, separately, for reference. The results from each are similar, with the only major difference being in the GWP100 (full life cycle) emissions estimates, owing to the fact that the Ecoinvent process includes a range of additional contributing sub-processes (e.g., trailer, shed, tyres, etc.).

Assuming use of Ecoinvent process “Diesel, burned in agricultural machinery {GLO}| diesel, burned in agricultural machinery | Alloc Def, U”:

Emissions estimates for harvesting 150 tons of beach-cast		Emissions estimates for harvesting 1 ton of beach-cast	
488	kg CO_2	3.26	kg CO_2
6.10	kg NO_X	0.041	kg NO_X
3.13E-03	kg NH_3	2.09E-05	kg NH_3
1456	kg CO_2 eq (GWP100)	9.7	kg CO_2 eq (GWP100)

Assuming use of USLCI process “Diesel, combusted in industrial equipment/US":

Emissions estimates for harvesting 150 tons of beach-cast		Emissions estimates for harvesting 1 ton of beach-cast	
493	kg CO_2	3.29	kg CO_2
9.64	kg NO_X	0.064	kg NO_X
n.a.	kg NH_3	n.a.	kg NH_3
585	kg CO_2 eq (GWP100)	3.90	kg CO_2 eq (GWP100)

Monetizing emissions from case study. The estimates above indicate that CO_2 emissions associated with the diesel consumption are approximately 3.3 kg CO_2 t^{-1} FW. The Swedish Transport Administration applies a social cost of carbon of SEK_{2017} 7 kg^{-1} CO_2 in transportation related CBAs (STA 2020), which indicates a total cost of about SEK_{2017} 2.06 million for the harvesting in the LOVA projects (3.3 × 89 287 t FW × 7), i.e., USD_{2018} 0.242 million, given adjustment for inflation with the Swedish consumer price index (OECD 2021) and conversion to USD with the mean USD/SEK currency rate for 2018 (8.6921, Sveriges Riksbank 2021). For NO_x and NH_4 emissions taking place in rural settings, STA (2020) applies social costs of SEK_{2017} 3.5 kg^{-1} NO_x (SEK_{2017} 1.50 related to effects of ground-level ozone plus SEK_{2017} 2 related to effects of marine eutrophication) and SEK_{2017} 8 kg^{-1} NH_3. For NO_x, this implies a total cost of about 0.05 kg NO_x t^{-1} FW × 89 287 t FW × SEK_{2017} 3.5 kg^{-1}

NO_x = SEK_{2017} 15 625 (USD_{2018} 1 833), and for NH_3 the corresponding calculation is 0.000021 × 89 287 × 8 = SEK_{2017} 15 (USD_{2018} 1.76).

S6. Harvest costs

Harvest cost figures for the LOVA projects are given in USD_{2018} in Table 3 in the article. Table S2 presents the original cost figures in SEK_{2018}. They were converted to USD_{2018} based on the mean USD/SEK currency rate for 2018 (8.6921, Sveriges Riksbank 2021).

Table S2. Harvest costs for the LOVA projects in SEK_{2018}.

	**Invoiced costs[a]**	**Volunteering through harvest vehicle driving[b]**	**Other volunteering[b]**	**Total costs**
Mean	134 945	32 430	79 604	246 979
SD	174 185	92 595	195 916	246 609
Median	86 839	0	46 100	188 831
Min	7 667	0	0	12 855
Max	942 490	408 500	528 000	962 490
Sum for 38 projects[c]	5 127 896	1 232 338	3 024 950	9 385 184
Sum for all 40 projects[d]	5 397 786	1 297 198	3 184 158	9 879 142
Cost t^{-1} FW harvest[e]	60	15	36	111
Cost kg^{-1} reduced PO_4-eq[f]	18	4.3	11	33

[a] Invoiced costs do not include 25 % VAT because it is assumed that harvesting activities do not displace other types of production, cf. Johansson and Kriström (2018).
[b] For volunteering, Swedish authorities apply a standard value of SEK 500 hr^{-1} for drivers of tractors and other vehicles, and SEK 200 hr^{-1} for all other activities.
[c] Cost data were reported for 38 of 40 projects.
[d] Mean costs were assumed for 2 projects with missing cost data.
[e] Based on a total harvest of 89 287 t FW, see Table 1 in the article.
[f] Based on a total nutrient removal of 301 949 kg PO_4-eq, see Table 1 in the article.

An alternative way of estimating average harvest costs per kg removed PO_4-eq is to use the nutrient concentration results in Franzén et al. (2019), see also Appendix S3. Dividing the cost t^{-1} FW in Table S2 by the mean nutrient concentration in Franzén et al. (2019), i.e., 3.96 kg PO_4-eq t^{-1} FW, results in SEK_{2018} 15 kg^{-1} removed PO_4-eq excl. volunteering (60 divided by 3.96) and SEK_{2018} 28 kg^{-1} removed PO_4-eq incl. volunteering (111 divided by 3.96), which is only a few SEK lower than the corresponding cost estimates in the last row of Table S2.

Substantial variability in harvest costs per ton harvest can be expected across beaches because of different harvest conditions due to the degree of accessibility for tractors, personnel skill and experience, depending on volunteering, etc. (Mossbauer et al. 2012), and there are even more potential sources of variability for costs expressed per kg removed PO_4-eq, because the projects' beach-cast sampling procedures also come into the picture as well as the natural variability in nutrient concentrations and the increased risk for the LOVA projects to make computational errors in the reporting.

For illustrating this, Table S3 reports the results of an analysis of costs per ton harvest and per kg removed PO_4-eq based on such costs for individual LOVA projects, where each project is equally weighted (=1). It is evident from the coefficient of variation that there is a substantial variability, in particular for the costs per kg removed PO_4-eq. This is to some extent explained by one project that reported very low nutrient concentrations: 0.52 kg N t^{-1} FW and 0.035 kg P t^{-1} FW, which is about one tenth of the mean concentrations found by Franzén et al. (2019). The fact that this project also had relatively high costs per ton harvest implies that the project experienced extremely high costs per kg removed PO_4-eq (i.e., the maximum values reported in Table S3). If this outlier is excluded from the analysis, the mean cost excluding volunteering is reduced from SEK_{2018} 88 to 58 kg^{-1} removed PO_4-eq, and the mean cost including volunteering is reduced from SEK_{2018} 225 to 150 kg^{-1} removed PO_4-eq. While the average situation for the LOVA projects indicates a cost including volunteering below

SEK_{2018} 100 kg^{-1} removed PO_4-eq (USD_{2018} 12), it is important that this cost can be considerably higher for individual projects.

Table S3. Harvest costs related to harvests and removed PO_4 equivalents for individual LOVA projects (SEK_{2018}), where each project is equally weighted (=1).

	**Cost excl. volunteering t^{-1} harvest FW**	**Cost incl. volunteering t^{-1} harvest FW**	**Cost excl. volunteering kg^{-1} removed PO_4-eq**	**Cost incl. volunteering kg^{-1} removed PO_4-eq**
Mean	138	274	88	225
SD	129	268	175	455
Median	86	159	38	69
Min	18	29	4	9
Max	518	960	950	2 302
Coefficient of variation	0.93	0.98	1.99	2.02
n	25	25	29	29

As a further illustration of variability, the results for the LOVA projects on Gotland can be compared to beach-cast harvest costs in other parts of Sweden. Wegenke (2016) reported results from a LOVA funded harvest project carried out by Borgholm Municipality at Köpingsvik on the island of Öland during 2014–2016. The results indicated a total harvest cost of SEK_{2018} 472 t^{-1} FW and SEK_{2018} 89 kg^{-1} removed PO_4-eq. This project included no volunteering but 10 % of the salary costs of a municipality official were included for project management, which implies that the most reasonable basis for comparison to the LOVA projects on Gotland is the costs including volunteering (which typically includes project management work). Two other examples are from the municipalities of Ystad and Trelleborg in the province of Scania in the southernmost part of the Swedish mainland. Nutrient removal data are not available for these examples, but beach-cast harvest costs in Ystad have amounted to about SEK 111–222 t^{-1} FW (Henrik Uthas, Ystad Municipality, pers. comm., 9

September 2019) and for Trelleborg, Blidberg et al. (2012) reported € 140 t^{-1} DW as a beach-cast removal cost, i.e., about € 30 t^{-1} FW if assuming the mean dry matter content of 24 % found by Franzén et al. (2019). While those cost estimates are found within the min-max interval of Table S3, they still illustrate the substantial variation. Gisselman (2014) used a macro algae harvest cost of SEK 1260 t^{-1} FW for the case of the bay of Burgsviken in Southern Gotland. This cost is higher than the max value in Table S3, but was for collecting free-floating macro algae, which can generally be expected to be more expensive than beach-cast harvest.

S7. Transaction costs

The costs for administrating the LOVA project system are identified as transaction costs. We delimit those costs to the administrative costs at the County Administrative Board of Gotland, whose officials are handling LOVA applications and reporting, and they also take part in informing project applicants and others about the LOVA system. LOVA project owners and other project participants can also be expected to incur administrative costs, but it is assumed that they are included in the volunteering reported in harvest costs (see Section 4.6 in the article). Salary costs related to administration of LOVA projects at the County Administrative Board of Gotland are available for the period of 2015–2018 in SwAM (2016, 2017, 2018, 2019), see Table S4. The reason for a relatively high salary cost per working hour in 2015 in comparison to 2016–2019 is unknown. Corresponding data for 2009–2014 are unfortunately not available from SwAM reporting. It is therefore assumed that the mean salary cost for 2015–2018, i.e., SEK_{2018} 461 750, can be used as an average for the whole period of 2009–2018, which gives a total cost for the whole period of SEK_{2018} to 4.62 million. The total number of LOVA project during this period was 54, of which 40 were about beach-cast

harvest, i.e., 74 %. This suggests an estimate of administrative costs related to beach-cast projects amounting to 4.62 × 0.74 = SEK_{2018} 3.42 million (USD_{2018} 0.393 million), given an assumption that the allocation of working hours was proportional across beach-cast harvest projects and other projects. One reason for the estimate not to be an underestimate is the fact that the number of approved beach-cast projects were greater in the period of 2015–2018 (n=25) than in 2009–2014 (n=15).

Table S4. Working hours and corresponding salary costs including social security fees for LOVA administration at the County Administrative Board of Gotland. Source: SwAM (2016, 2017, 2018, 2019).

**Year**	**Working hours**	**Salary costs in SEK (nominal)**	**Salary costs in SEK_{2018}[a]**
2015	842	731 000	766 000
2016	704	339 000	352 000
2017	708	343 000	350 000
2018	792	379 000	379 000
[a] Adjusted for inflation by the consumer price index for Sweden (OECD 2021).			

References

Ahtiainen, H., J. Artell, M. Czajkowski, B. Hasler, L. Hasselström, A. Huhtala, J. Meyerhoff, J. Smart, et al. 2014. Benefits of meeting nutrient reduction targets for the Baltic Sea – a contingent valuation study in the nine coastal states. *Journal of Environmental Economics and Policy* 3: 278-305.

Blidberg, E., F. Gröndahl, B. Cahill, J. Koreiviene, O. Belous, and D. Shabayeva. 2012. Macroalgae harvesting and cultivation. In *SUBMARINER Compendium: An Assessment of Innovative and Sustainable Uses of Baltic Marine Resources*, ed. A. Schultz-Zehden, and M. Matczak. Gdansk, Poland: Maritime Institute in Gdansk. ISBN 978-83-62438-14-3.

Charlier, R. H., P. Morand, C. W. Finkl, and A. Thys. 2007. Green tides on the Brittany coast. *Environmental Research, Engineering and Management* 3: 52-59.

Cruz, C. J., J. J. Muñoz-Perez, M. I. Carrasco-Braganza, P. Poullet, P. Lopez-Garcia, A. Contreras, and R. Silva. 2020. Beach cleaning costs. *Ocean and Coastal Management* 188: 105118. https://doi.org/10.1016/j.ocecoaman.2020.105118.

Czajkowski, M., H. Ahtiainen, J. Artell, W. Budziński, B. Hasler, L. Hasselström, J. Meyerhoff, T. Nõmmann, et al. 2015. Valuing the commons: An international study on the recreational benefits of the Baltic Sea. *Journal of Environmental Management* 156: 209-217.

ECB. 2021. ECB euro reference exchange rate. European Central Bank, Frankfurt, Germany.

Franzén, D., E. Infantes, and F. Gröndahl, F. 2019. Beach-case as biofertiliser in the Baltic Sea region: Potential limitations due to cadmium-content. *Ocean and Coastal Management* 169: 20-26.

Gisselman, F. 2014. Economic assessment of harvesting and removing macro algae and reed as a eutrophication mitigation method: A cost-benefit analysis using an ecosystem service approach applied on Burgsviken, Gotland. Master Thesis, Department of Economics, Umeå University, Umeå, Sweden.

Gren, I.-M., T. Söderqvist, and F. Wulff. 1997. Nutrient reductions to the Baltic Sea: Ecology, costs and benefits. *Journal of Environmental Management* 51: 123-143.

Hasselström, L., J.-B. Thomas, J. Nordström, G. Cervin, G. M. Nylund, H. Pavia, and F. Gröndahl. 2020. Socioeconomic prospects of a seaweed bioeconomy in Sweden. *Scientific Reports* 10: 1610. https://doi.org/10.1038/s41598-020-58389-6.

HELCOM, 2006. Development of tools for assessment of eutrophication in the Baltic Sea. Baltic Sea Environment Proceedings No. 104. Helsinki Commission, Helsinki, Finland.

HELCOM, 2015. Updated Fifth Baltic Sea pollution load compilation (PLC-5.5). Baltic Sea Environment Proceedings No. 145. Helsinki Commission, Helsinki, Finland.

Hyytiäinen, K., L. Ahlvik, H. Ahtiainen, J. Artell, A. Huhtala, and K. Dahlbo. 2015. Policy goals for improved water quality in the Baltic Sea: When do the benefits outweigh the costs?

Environment and Resource Economics 61: 217–241. https://doi.org/10.1007/s10640-014-9790-z

Kinell, G., T. Söderqvist, R. Elmgren, J. Walve, and F. Franzén. 2012. Cost-benefit analysis in a framework of stakeholder involvement and integrated coastal zone modeling. CERE Working Paper 2012:1, Umeå University and Swedish University of Agricultural Sciences, Umeå, Sweden.

Johansson, P.-O., and B. Kriström. 2018. *Cost-Benefit Analysis*. Cambridge, UK: Cambridge University Press.

Johnston, R. J., K. J. Boyle, M. L. Loureiro, S. Navrud, and J. Rolfe. 2021. Guidance to enhance the validity and credibility of environmental benefit transfer. *Environmental and Resource Economics* 79: 575–624. https://doi.org/10.1007/s10640-021-00574-w

Larsson, U., A. Sjösten, S. Hajdu, S. Nyberg, M. Sandberg, and J. Walve. 2011. Himmerfjärden 2010. Rapport till SYVAB. Technical Report No. 48, Department of Systems Ecology, Stockholm University, Stockholm.

Marineregions.org. 2020a. Marine Gazetteer Placedetails for Finnish Exclusive Economic Zone. Retrieved 24 April 2020, from http://www.marineregions.org/eezdetails.php?mrgid=5676

Marineregions.org. 2020b. Marine Gazetteer Placedetails for Swedish Exclusive Economic Zone. Retrieved 24 April 2020, from http://www.marineregions.org/eezdetails.php?mrgid=5694

Mossbauer, M., I. Haller, S. Dahlke, and G. Schernewski. 2012. Management of stranded eelgrass and macroalgae along the German Baltic coastline. *Ocean & Coastal Management* 57: 1-9.

Mossone, P., I. Guala, and S. Simeone. 2019. Posidonia banquettes on the Mediterranean beaches: To what extent do local administrators' and users' perceptions correspond? In *Planning, nature and ecosystem services*, ed. C. Gargiulo and C. Zoppi, 225-234. Naples, Italy: FedOAPress. ISBN: 978-88-6887-054-6. doi: 10.6093/978-88-6887-054.6

Mouat, J., R. Lopez, and H. Bateson. 2010. Economic impacts of marine litter. Report, KIMO (Local Authorities International Environmental Organisation), Lerwick, UK.

Nieminen, E., H. Ahtiainen, C.-J. Lagerkvist, and S. Oinonen. 2019. The economic benefits of achieving Good Environmental Status in the Finnish marine waters of the Baltic Sea. *Marine Policy* 99: 181-189.

OECD. 2021. Consumer price index, CPI 01-12. OECD, Paris.

Ofori, R.O., and M. D. Rouleau. 2020. Willingness to pay for invasive seaweed management: Understanding how high and low income households differ in Ghana. *Ocean and Coastal Management* 192: 105224.

Östberg, K., L. Hasselström, and C. Håkansson. 2012. Non-market valuation of the coastal environment: Uniting political aims, ecological and economic knowledge. *Journal of Environmental Management* 110, 166-178.

Risén, E., J. Nordström, M. E. Malmström, and F. Gröndahl. 2017. Non-market values of algae beach-cast management: Study site Trelleborg, Sweden. *Ocean & Coastal Management* 140: 59-67.

Scharin, H., S. Ericsdotter, M. Elliott, R. K. Turner, S. Niiranen, T. Blenckner, K. Hyytiäinen, L. Ahlvik, H. Ahtiainen, J. Artell, L. Hasselström, T. Söderqvist, and J. Rockström. 2016. Processes for the sustainable stewardship of marine environments. *Ecological Economics* 128: 55–67.

STA. 2020. Analysmetod och samhällsekonomiska kalkylvärden för transportsektorn, ASEK 7.0. Swedish Transport Administration, Borlänge, Sweden. Retrieved 26 November 2020, from www.trafikverket.se/asek

Sveriges Riksbank, 2021. Årsgenomsnitt valutakurser. Sveriges Riksbank, Stockholm.

SwAM. 2016. Åtgärder för havs- och vattenmiljö: Rapportering av användningen av anslag 1:12 under 2015. Rapport 2016-04-18, Swedish Agency for Marine and Water Management, Göteborg, Sweden.

SwAM. 2017. Åtgärder för havs- och vattenmiljö: Rapportering av användningen av anslag 1:12 under 2016. Rapport 2017-03-31, Swedish Agency for Marine and Water Management, Göteborg, Sweden.

SwAM. 2018. Återrapportering av användning av anslag 1:11: Åtgärder för havs- och vattenmiljö under 2017 enligt regleringsbrev för budgetåret 2017. Report to the Ministry of Environment and Energy dated 27 March 2018. Swedish Agency for Marine and Water Management, Göteborg, Sweden.

SwAM. 2019. Återrapportering av användning av anslag 1:11: Åtgärder för havs- och vattenmiljö under 2018 enligt regleringsbrev för budgetåret 2018. Report to the Ministry of Environment and Energy dated 29 March 2019. Swedish Agency for Marine and Water Management, Göteborg, Sweden.

VISS. 2020. Vatteninformationssystem Sverige, Skagerrak. Swedish Water Authorities. Retrieved 24 April 2020, from https://viss.lansstyrelsen.se/MarineRegions.aspx?marineRegionEUID=ANS-SE-AA-B_Skagerrak

Wegenke, J. 2016. LOVA släk – lyft näringen från havet. Report, Borgholm Municipality, Borgholm, Sweden. Retrieved 5 August 2020, from https://www.borgholm.se/wp-content/uploads/2018/01/LOVA-SL%C3%84K-Lyft-n%C3%A4ringen-fr%C3%A5n-havet-Slutrapport.pdf

World Bank, 2021. World Development Indicators: Consumer price index, FP.CPI.TOTL. The World Bank, New York.

Yang, Y., J. Boncoeur, S. G. Liu, and P. Nyvall-Collen. 2018. Economic assessment and environmental management of green tides in the Chinese Yellow Sea. *Ocean and Coastal Management* 161: 20–30.

Zielinski, S., C. M. Botero, and A. Yanes. 2019. To clean or not to clean? A critical review of beach cleaning methods and impacts. *Marine Pollution Bulletin* 139: 390–401. https://doi.org/10.1016/j.marpolbul.2018.12.027
